# Revisiting the formalism of equivalent uniform dose based on the linear-quadratic and universal survival curve models in high-dose stereotactic body radiotherapy

**DOI:** 10.1007/s00066-020-01713-w

**Published:** 2020-11-27

**Authors:** Mark Ka Heng Chan, Chi-Leung Chiang

**Affiliations:** 1 Department of Radiotherapy, West German Cancer Center, University Hospital Essen, Essen, Germany; 2 Department of Radiation Oncology, Karl-Lennert-Krebscentrum Nord, University Medical Center Schleswig-Holstein, Campus Kiel, Arnold-Heller-Straße 3, Haus 50, 24105 Kiel, Germany; 3 Department of Clinical Oncology, The University of Hong Kong, Hong Kong, Hong Kong S.A.R., China

**Keywords:** Equivalent uniform dose, Linear-quadratic model, Universal survival curve, Stereotactic body radiotherapy, Lung cancers

## Abstract

**Purpose:**

To examine the equivalent uniform dose (EUD) formalism using the universal survival curve (USC) applicable to high-dose stereotactic body radiotherapy (SBRT).

**Materials and methods:**

For nine non-small-cell carcinoma cell (NSCLC) lines, the linear-quadratic (LQ) and USC models were used to calculate the EUD of a set of hypothetical two-compartment tumor dose–volume histogram (DVH) models. The dose was varied by ±5%, ±10%, and ±20% about the prescription dose (60 Gy/3 fractions) to the first compartment, with fraction volume varying from 1% and 5% to 30%. Clinical DVHs of 21 SBRT treatments of NSCLC prescribed to the 70–83% isodose lines were also considered. The EUD of non-standard SBRT dose fractionation (EUD_SBRT_) was further converted to standard fractionation of 2 Gy (EUD_CFRT_) using the LQ and USC models to facilitate comparisons between different SBRT dose fractionations. Tumor control probability (TCP) was then estimated from the LQ- and USC-EUD_CFRT_.

**Results:**

For non-standard SBRT fractionation, the deviation of the USC- from the LQ-EUD_SBRT_ is largely limited to 5% in the presence of dose variation up to ±20% to fractional tumor volume up to 30% in all NSCLC cell lines. Linear regression with zero constant yielded USC-EUD_SBRT_ = 0.96 × LQ-EUD_SBRT_ (*r*^*2*^ = 0.99) for the clinical DVHs. Converting EUD_SBRT_ into standard 2‑Gy fractions by the LQ formalism produced significantly larger EUD_CFRT_ than the USC formalism, particularly for low $\alpha /\beta$ ratios and large fraction dose. Simplified two-compartment DVH models illustrated that both the LQ- and USC-EUD_CFRT_ values were sensitive to cold spot below the prescription dose with little volume dependence. Their deviations were almost constant for up to 30% dose increase above the prescription. Linear regression with zero constant yielded USC-EUD_CFRT_ = 1.56 × LQ-EUD_CFRT_ (*r*^*2*^ = 0.99) for the clinical DVHs. The clinical LQ-EUD_CFRT_ resulted in median TCP of almost 100% vs. 93.8% with USC-EUD_CFRT_.

**Conclusion:**

A uniform formalism of EUD should be defined among the SBRT community in order to apply it as a single metric for dose reporting and dose–response modeling in high-dose-gradient SBRT because its value depends on the underlying cell survival model and the model parameters. Further investigations of the optimal formalism to derive the EUD through clinical correlations are warranted.

**Electronic supplementary material:**

The online version of this article (10.1007/s00066-020-01713-w) contains supplementary material, which is available to authorized users.

## Introduction

Dose distributions for stereotactic body radiotherapy (SBRT) are often heterogeneous when treatment doses are prescribed to lower isodose lines (IDL), i.e., from ~70% to 90%, to create more rapid dose fall off [1–3]. The difficulty is in characterizing and reporting these dose distributions within the tumor that are typically delivered at a high dose per fraction [4].

One of the most useful concepts for assessing the radiobiological impact of the non-uniform dose distribution is the equivalent uniform dose (EUD) [5]. EUD converts a heterogeneous dose distribution into a single dose measure, taking into account the non-linear dose–response relationship by incorporating the linear-quadratic (LQ) formalism.

On the other hand, it is questionable how far the EUD would be applicable in SBRT considering its origin from the LQ model. Its validity beyond the conventional dose range is subject to the same scrutiny as the LQ-based biological effective dose used in SBRT [6, 7]. Although the LQ model has been proven to be robust at describing and predicting radiation effects for “conventional” doses, i.e., of around 2 Gy per fraction, its use for higher values of dose per fraction remains controversial [8, 9]. As pointed out by McKenna and Ahmad [10], 2 Gy often corresponds to a point on the initial linear region of the cell survival curve or that just preceding the shoulder region. Beyond the shoulder, the LQ model underestimates the cell survival, predicting a quadratic dependence on the dose for negative log cell survival as the dose delivered increases. However, the survival curve slope has been observed to approach a constant with increasing dose in experimentally measured data [6]. Binary misrepair models account for this change in the cell survival curve slope from near quadratic to near linear by suggesting that there is a change from restitution to binary misrepair as the method by which double-strand breaks (DSB) are removed from DNA [11]. Hanin and Zaider also provide a detailed microdosimetry treatment of LQ formalisms in which they attribute the failure of LQ formalisms at high doses to saturation effects and/or the assumption of a Poisson distribution for the lesions [12].

Park et al. proposed a universal survival curve (USC) as an alternative to the LQ model to model cell killing in the typical dose range of lung SBRT [6]. The USC is produced by hybridizing two classical radiobiological models: LQ and multi-target single hit (MTSH) [13]. At high doses, the MTSH model predicts a linear dependence of the negative log cell survival on dose, as opposed to the quadratic dependence in the LQ model. Park et al. found that the USC model significantly improved the goodness of fit to in-vitro data for the H460 cell line (non-small-cell carcinoma, NSCLC) compared to the LQ model. Andisheh et al. later confirmed the better fit to the H460 cell line with the USC and further showed superior fits to other small-cell lung cancer (SCLC) cell lines (U1690 and NCI-H841) [14]. These in-vitro cell survival models have been extrapolated to in-vivo treatment outcomes in SBRT of NSCLC but largely ignoring the non-uniform three-dimensional dose profiles of individual patients [15, 16].

With the many models of and fits to in-vitro cell survival data over the extended dose ranges that have become available recently [10, 17–19], it would be of interest to revisit the EUD formalism. In this work, we aim to express EUD in terms of the USC model and make comparisons with the LQ-based EUD values.

## Materials and methods

### The LQ formalism and the universal survival curve for clonogenic cell survivals

The LQ formalism was originally a low-dose approximation to in vitro cell survival data. In LQ formalism the relationship between surviving cell fraction, SF, and dose, D, is given by [20]

1 $$\text{SF}(\text{D})=\text{e}^{-\left(\alpha \cdot \text{D}+\beta \cdot \text{D}^{2}\right)}$$

where $\alpha$ and $\beta$ are defined as the dose-rate-independent inactivation coefficients for lethal cell damage resulting from one ionizing event and two independent interacting ionizing events, respectively [11]. For radiotherapy treatment of total dose *D* delivered in *n* fractions with a dose per fraction of *d*:

2 $$\text{SF}(n\cdot d)=\text{e}^{-n\left(\alpha \cdot d+\beta \cdot d^{2}\right)}$$

On the other hand, the universal survival curve (USC) interpolates between these two models so that [6]

3 $$\text{SF}(n\cdot d)=\begin{cases} \text{e}^{-n\left(\alpha \cdot \text{D}+\beta \cdot \text{D}^{2}\right)} \quad \mathrm{if}\ \mathrm{d}\leq \mathrm{d}_{\mathrm{T}}\\ \text{e}^{-\frac{n}{\text{D}_{0}}\left(d-\text{D}_{q}\right)}\quad \mathrm{if}\ \mathrm{d}>\mathrm{d}_{\mathrm{T}} \end{cases}$$

where −1/D_0_ is the final slope of the MTSH model, D_0_ is the dose in Gy that reduces survival to e^−1^, and D_q_ is the x‑intercept of the asymptote for the MTSH model at *d* ≫ D_0_. We use d_T_ to indicate the dose per fraction at which the transition between these two models occurs. As the curve is continuous it is considered to be differentiable at d_T_ so that the five parameters in the above equations—$\alpha$,$\beta$, D_0_, D_q_, and d_T_—can be reduced to three parameters as follows:

4 $$\begin{aligned} \beta &= \frac{(1-\alpha \cdot \text{D}_{0})^{2}}{4\text{D}_{0}\cdot \text{D}_{q}}\\ \mathrm{d}_{\mathrm{T}} &= \frac{2\text{D}_{q}}{1-\alpha \cdot \text{D}_{0}} \end{aligned}$$

The USC model therefore has one more parameter than the LQ model.

### The equivalent uniform dose: the LQ and USC formalisms

For an inhomogeneous dose distribution delivered to a tumor volume via a fractionated regime, Niemierko [5] suggests that there should be an equivalent uniform dose distribution that will yield the same surviving cell fraction. Mathematically expressed:

5 $$\text{SF}(\text{EUD})=\sum _{i}^{N}v_{i}\cdot \text{SF}\left(\text{D}_{i}\right)$$

where D_i_ is the total dose to tumor subvolume $v_{i}$, and *N* is the number of subvolumes. The sum of all of the subvolumes is unity. To incorporate the dose-per-fraction dependence of cell killing, Jones and Hoban combined the concept of biological equivalent dose (BED) with the EUD, and introduced the biological equivalent uniform dose (BEUD) [21]. The BED allows the comparison between radiotherapy treatments of different fractionation schemes and is related to *SF* by

6 $$\text{SF}_{\text{BED}}=\text{e}^{-\alpha \cdot \text{BED}}$$

Equating Eq. 6 with Eq. 2, Eq. 3 gives the BED for all dose ranges

7 $$\text{BED}=\begin{cases} n\cdot d\left(1+\frac{d}{\alpha /\beta }\right)\quad \mathrm{if}\ \mathrm{d}\leq \mathrm{d}_{\mathrm{T}}\\ \frac{n}{\alpha \cdot \text{D}_{0}}\left(d-\text{D}_{q}\right)\quad \mathrm{if}\ \mathrm{d}>\mathrm{d}_{\mathrm{T}} \end{cases}$$

Eq. 5 can then be rewritten in terms of BED and BEUD as

8 $$\text{e}^{-(\alpha \cdot \text{EUBED})}=\sum_{i}^{N}v_{i}\cdot \text{e}^{-\left(\alpha \cdot \text{BED}_{i}\right)}$$

Further substituting the LQ-BED (Eq. 7a) into Eq. 8 yields

9 $$\text{e}^{-\left(\alpha \cdot \text{EUD}+\frac{\beta \cdot \text{EUD}^{2}}{n}\right)}=\sum _{i}^{N}v_{i}\cdot \text{e}^{-\left(\alpha \cdot \text{D}_{i}+\frac{\beta \cdot \text{D}_{i}^{2}}{n}\right)}$$

The solution of Eq. 8 gives the following expression for EUD:

10 $$\text{EUD}=\frac{-n\alpha }{2\beta }+\frac{n}{2\beta }\left\{\alpha ^{2}-\frac{4\beta }{n}\times \ln \left[\sum _{i}^{N}v_{i}\cdot \text{e}^{\left(-\alpha \cdot \text{D}_{i}-\frac{\beta \cdot \text{D}_{i}^{2}}{n}\right)}\right]\right\}^{1/2}$$

Originally, Niemierko [5] expressed the EUD in terms of the surviving fraction of clonogenic cells after a dose 2 Gy. In theory, Eq. 5 can be modified to accommodate any model of the dose–response relationship. Substituting the USC model (Eq. 7b) into Eq. 5 yields the following expression:

11 $$\text{e}^{-\left(\frac{\text{EUD}-n\text{D}_{q}}{\text{D}_{0}}\right)}=\sum _{i}^{N}v_{i}\cdot \text{e}^{-\left(\frac{\text{D}_{i}-n\text{D}_{q}}{\text{D}_{0}}\right)}$$

After a rearrangement of terms EUD is given by:

12 $$\text{EUD}=-\text{D}_{0}\ln \left(\sum _{i}^{N}v_{i}\cdot \text{e}^{\frac{-\text{D}_{i}}{\text{D}_{0}}}\right) \mathrm{for}\ \mathrm{d}>\mathrm{d}_{\mathrm{T}}$$

Therefore, the EUD expression based on the USC model at d > d_T_ simply reduces to the original functional form given by Niemierko [5] based on the single-target (ST) model that describes cell killing as exponential, i.e., $SF(D)=\exp (-D/D_{0})$, as the initial shoulder, and associated quasi-threshold dose (D_q_) is no longer present. For a given dose distribution, the USC-EUD depends only on D_0_. At d_T_ and below, the EUD formalism remains unchanged because the USC and LQ formalism are the same. The EUD in Eq. 10 (LQ) and Eq. 12 (USC) are both defined as the uniform dose given in the same number of fractions as the original inhomogeneous dose. To translate the EUD in SBRT schedule (i.e., EUD_SBRT_) into a different schedule of equivalent biologic effect, the BED concept in Eq. 7 is applied,

13 $$\text{EUD}_{\text{CFRT}}=\text{EUD}_{\text{SBRT}}\frac{\left(1+\frac{\text{EUD}_{\text{SBRT}}}{n_{\text{SBRT}}\frac{\alpha}{\beta}}\right)}{\left(1+\frac{d_{\text{ref}=2\,\text{Gy}}}{\frac{\alpha}{\beta}}\right)}\quad \text{with the LQ model}$$

14 $$\text{EUD}_{\text{CFRT}}=\frac{1}{\alpha \text{D}_{0}} \frac{\left(\text{EUD}_{\text{SBRT}}-n_{\text{SBRT}}\text{D}_{q}\right)}{\left(1+\frac{d_{\text{ref}=2\,\text{Gy}}}{\frac{\alpha}{\beta}}\right)}\quad \text{with the USC for}\ \mathrm{d}>\mathrm{d}_{\mathrm{T}}$$

where EUD_CFRT_ specifies the total EUD delivered in a conventional-fractionation radiotherapy (CFRT) schedule with standard 2‑Gy fractions for the equivalent effect. For convenient notation, the EUD computed with the LQ (Eq. 10) and USC (Eq. 12) formalisms for SBRT schedule are denoted as LQ-EUD_SBRT_ and USC-EUD_SBRT_. Similar notations apply to EUD normalized to 2‑Gy fractions, i.e., USC-EUD_CFRT_ and LQ-EUD_CFRT_.

### Model parameters of the LQ and USC and sensitivity of model parameters

For this study, we used fitted parameters for nine selected non-small-cell lung cancer (NSCLC) cell lines documented in the work of Carmichael et al. [22]. These were comprised of three adenocarcinoma (NCI-H23, NCI-H522, NCI-H538), two adenosquamous (NCI-H596), one squamous cell (NCI-H226), one mesothelioma (NCI-H290), and two large cell anaplastic (NCI-H460 and NCI-H661) cell lines. The cell survival parameters of these cell lines are listed in Table 1. Using Eq. 4b values for the transition dose, d_T_, were calculated and found to be in the range of 4.0–7.1 Gy with a mean of 5.8 Gy.

**Table 1:** Fitting cell survival curve parameters of nine selected non-small-cell lung cancer cell lines obtained from [22]

Cell type	Cell lines	d_T_	D_q_	D_0_	$\alpha$	$\beta$	$\alpha /\beta$	*n*
Large cell anaplastic	NCI-H460	5.35	1.53	1.00	0.43	0.047	9.15	7
	NCI-H661	7.10	3.15	1.40	0.08	0.045	1.78	9.5
Adenocarcinoma	NCI-H23	4.03	0.19	1.02	0.89	0.010	89.00	1.2
	NCI-H522	6.74	2.11	1.10	0.34	0.034	10.00	6.8
	NCI-H358	5.64	1.91	1.20	0.27	0.046	5.87	4.9
Mesothelioma	NCI-H290	6.56	0.76	1.30	0.59	0.012	49.17	1.8
Adenosquamous	NCI-H596	5.88	2.23	1.35	0.18	0.046	3.91	5.2
	NCI-H647	5.42	1.49	1.50	0.30	0.030	10.00	2.7
Squamous	NCI-H226	5.64	1.65	1.60	0.26	0.031	8.39	2.8

*d*_*T*_ transition dose of the linear-quadratic-linear (USC) cell survival model; *−1/D*_*0*_ final slope of the multi-target single-hit model; *D*_*q*_ x-intercept of asymptote for the multi-target model

### Model and clinical dose distributions

To quantify the behaviors of the USC- and LQ-EUD with dose variation to different target subvolumes we assumed a tumor comprising two subvolumes, with a fractional volume of 1% and 5% to 30% in increments of 5% in the first compartment. The dose in the first subvolume was varied by ±5%, ±10%, and ±20% about the prescription dose of 20 Gy for three fractions to simulate hot and cold spots in the hypothetical tumor dose–volume histogram (DVH) models. The dose was fixed to the prescribed dose in the second subvolume with the fractional volume according to the first.

Clinical data comprising 21 DVHs of the gross tumor volume (GTV) were obtained from SBRT lung treatments using CyberKnife (Accuray, Sunnyvale, CA, USA), as described in [23]. For all clinical plans, the GTV coincided with the clinical target volume and was expanded by 3 mm to produce the planning target volume (PTV). The treatment regime was a total dose of 60 Gy delivered in three 20-Gy fractions prescribed to IDLs in the range 70% to 83%. A ray-tracing dose algorithm with equivalent path length correction was used for dose optimization and prescription. This dose regime was estimated to be approximately equal to 54 Gy in three fractions if a Monte Carlo dose engine were used in place of the ray tracing [24]. Since different dose fractionation schemes are adapted in lung SBRT, the EUD_SBRT_ and EUD_CFRT_ values were also re-calculated to simulate the other common treatment regime of 48 Gy in four fractions to evaluate this effect.

Analysis of the results for both fractionation schemes showed that none of these cell lines had a transition dose, d_T_, that was lower than the coldest dose point within the PTV. Therefore, use of the LQ (Eq. 10) or the USC formalism (Eq. 12) alone was again sufficient for calculating the EUD in all the dose distributions analyzed.

To assess the clinical impact of the EUD formalisms, the local progression-free survival (LPFS) at 30 months was estimated according to the logistic model with published parameters of *D*_*50*_ = 84.5 Gy and γ = 1.5 given by Martel et al. [25], where *D*_*50*_ is the dose to achieve 50% of LPFS and γ is the slope at *D*_*50*_.

## Results

### Two-bins model dose volume histograms (DVHs)

**Fig. 1:**
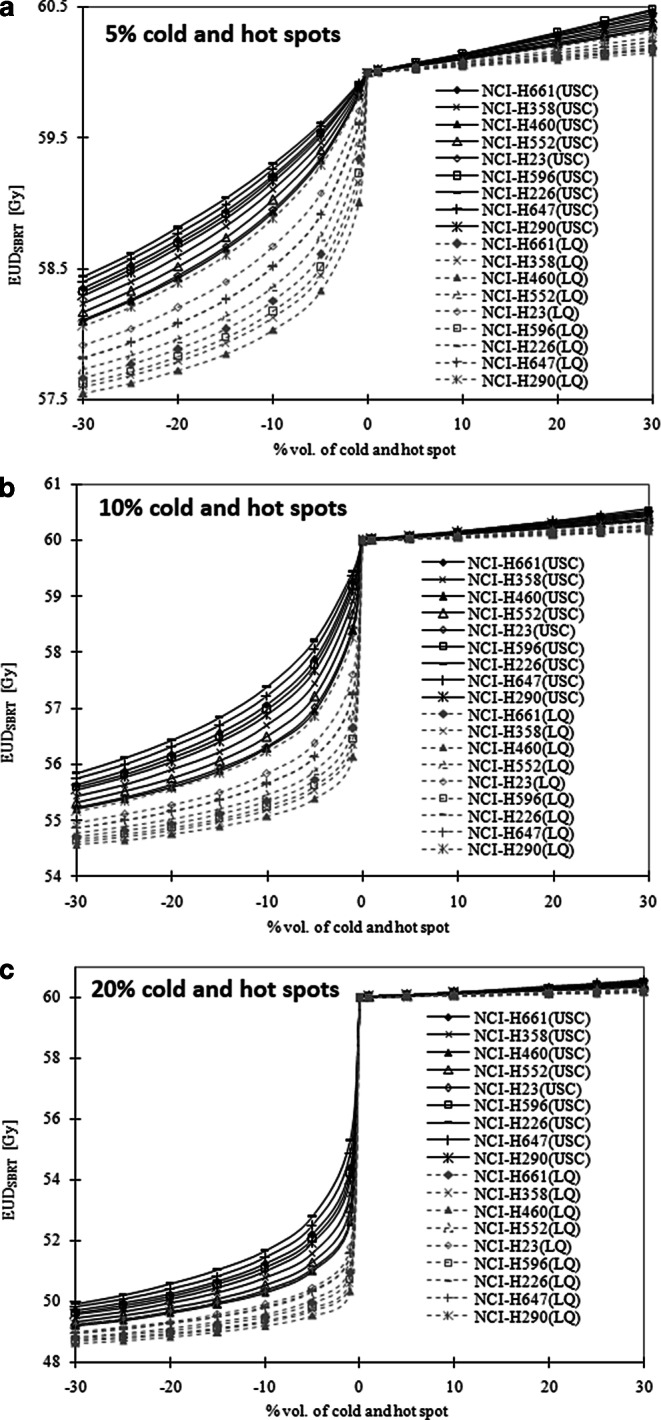
Calculated values of the USC- and LQ-EUD_SBRT_ (20 Gy × 3 fractions) for 5% (**a**), 10% (**b**) and 20% (**c**) dose variation as a function of percentage volume (% vol.) of cold and hot spot for nine NSCLC cell lines. (Note: the negative % vol. corresponds to cold spot and positive % vol. otherwise.)

Fig. 1 shows the EUD_SBRT_ for 5%, 10%, and 20% over- and underdosage to different target subvolumes for all studied cell lines. Compared to the LQ-EUD formalism the USC-EUD formalism results in larger values of EUD_SBRT_ for both over- and underdosage from 5 to 20% to all target subvolumes (paired *t*-tests; *p* < 0.05). Both the USC- and LQ-EUD_SBRT_ decreased non-linearly with increasing underdosage (dose cold spot) but approximately linearly with increasing overdosage (dose hot spot). Among the cell lines under examination, the NCI-H460 of the large cell anaplastic cell lines (LCC) was found to be most sensitive to dose cold spot with both formalisms. For the worst case scenario in which 30% of the target volume is underdosed by 20%, the EUD_SBRT_ decreased by up to 18.0% for the USC formalism and by up to 19.0% for the LQ formalism with reference to a uniform dose of 60 Gy. The least sensitive cell lines were the NCI-H226 of the squamous cell carcinoma (SCC) in the USC formalism and the NCI-H290 of the mesothelioma in the LQ formalism, with maximum decreases in the EUD_SBRT_ of 2.6% and 3.2%, respectively. Overall, the effect of dose boost to the target’s subvolume was fairly limited considering the small increase in EUD values (<1%).

**Fig. 2:**
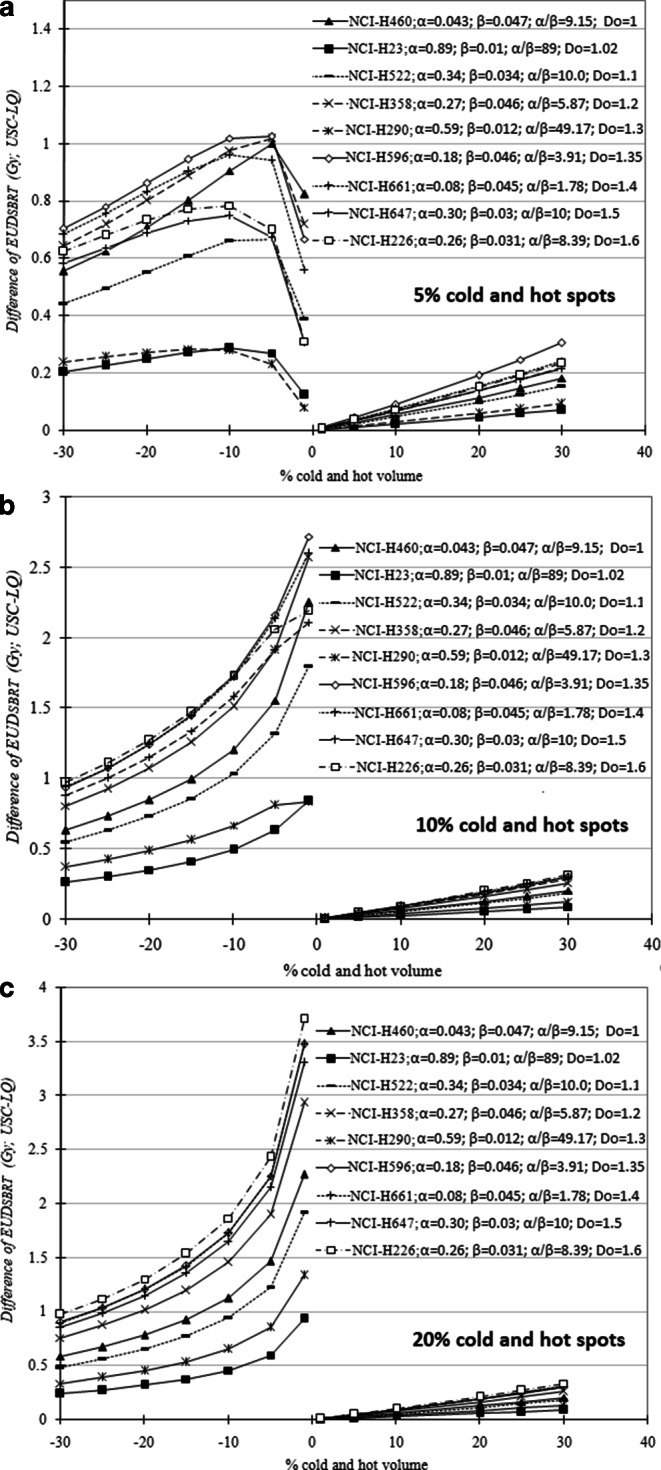
Deviations of the USC-EUD_SBRT_ from LQ-EUD_SBRT_ for 5% (**a**), 10% (**b**), and 20% (**c**) dose variations as a function of the % volume of cold and hot spot for nine NSCLC cell lines

Fig. 2 shows that the differences of EUD_SBRT_ between the USC and LQ formalisms are clearly different for dose cold spots and hot spots. They are larger for underdosage and smaller for overdosage. The largest deviation between the two formalisms (~3.7 Gy, or 7.2%) was observed in dose distributions where small target subvolumes have a large dose cold spot. It is worth noting that for small dose cold spots (~5%) the difference of EUD_SBRT_ between the two formalisms initially increases with increasing underdosed subvolumes from 1% and then reduces until about 5 to 10%, depending on cell line.

EUD values after normalization to 2 Gy per fraction (i.e., EUD_CFRT_) according to Eq. 13 and Eq. 14 are given, in part, in Table 2. These results are the reverse of the effect of using different formalisms in calculating EUD_SBRT_, as LQ-EUD_CFRT_ is larger than USC-EUD_CFRT_ in all cases (*p* < 0.05).

**Table 2:** EUD calculated and then corrected to 2‑Gy fractions by the USC and the LQ model. In the two-bin model DVH, the dose is varied by 10% and 20% of the prescription dose to 1% to 30% target subvolume. (Partial results were given due to space limit)

Cell lines	% dose variation		% vol. variation					
			−30%	−20%	−10%	10%	20%	30%
NCI-H661	10%	USC	205.6	207.9	211.6	224.5	225.2	226.0
		LQ	289.8	292.2	296.2	346.5	347.2	348.0
	20%	USC	180.6	183.0	187.0	224.5	225.2	226.0
		LQ	233.0	235.3	239.4	346.5	347.2	348.0
NCI-H596	10%	USC	137.4	138.8	141.2	149.8	150.2	150.7
		LQ	204.5	206.0	208.6	243.0	243.5	244.0
	20%	USC	121.2	122.7	125.2	149.8	150.3	150.7
		LQ	166.1	167.6	170.1	243.0	243.5	244.0
NCI-H358	10%	USC	115.7	116.8	118.6	126.5	126.8	127.2
		LQ	176.5	170.2	168.2	197.5	197.9	198.2
	20%	USC	101.9	103.0	104.9	126.5	126.8	127.2
		LQ	136.7	137.8	139.8	197.5	197.9	198.2
NCI-H226	10%	USC	102.1	103.3	105.1	110.5	110.9	111.3
		LQ	140.9	142.2	144.3	164.3	164.7	165.1
	20%	USC	90.6	91.9	94.0	110.5	110.9	111.3
		LQ	116.4	117.7	119.8	164.3	164.7	165.1
NCI-H460	10%	USC	95.4	96.2	97.5	104.8	105.0	105.3
		LQ	133.8	134.6	135.9	157.1	157.3	157.6
	20%	USC	84.0	84.8	86.1	104.8	105.0	105.3
		LQ	110.6	111.3	112.7	157.1	157.3	157.6
NCI-H522	10%	USC	107.3	108.3	110.0	118.0	118.3	118.6
		LQ	129.0	129.9	131.6	150.3	150.5	150.9
	20%	USC	94.0	95.0	96.7	118.0	118.3	118.6
		LQ	107.0	107.9	109.6	150.3	150.5	150.9
NCI-H290	10%	USC	67.1	67.7	68.7	72.8	73.0	73.2
		LQ	72.8	73.5	74.6	81.3	81.5	81.7
	20%	USC	59.6	60.2	61.4	72.8	73.0	73.2
		LQ	63.1	63.8	64.9	81.3	81.5	81.7
NCI-H23	10%	USC	58.8	59.3	60.0	64.1	64.2	64.4
		LQ	64.8	65.3	66.0	72.0	72.1	72.3
	20%	USC	52.4	52.8	53.6	64.1	64.2	64.4
		LQ	56.7	57.2	57.9	72.0	72.1	72.3

Note: the positive percentage volume (% vol.) indicates an increase of dose and negative % vol. otherwise.

### Clinical DVHs

**Fig. 3:**
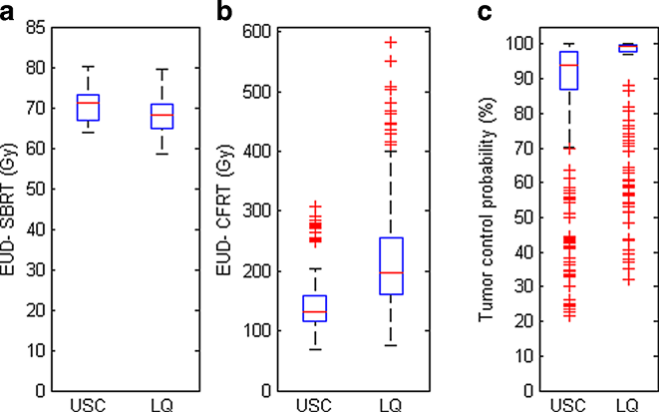
Distributions of **a** EUD_SBRT_, **b** EUD_CFRT_ with dose-per-fraction correction to 2‑Gy equivalents by the USC (outliers represent the NCI-661 cell line) and the LQ (outliers represent the NCI-661 and NCI-569 cell lines) models, and **c** tumor control probability (outliers represent the NCI-23 and -290 cell lines) that were calculated from the clinical dose–volume histograms. The box represents the interquartile (IQ) range and the whiskers indicate the highest and lowest values that are no greater than 1.5 × IQ range. Outliers represent values beyond 1.5 × IQ range

Results for clinical DVHs are summarized in Fig. 3. Using LQ and USC models for the EUD_SBRT_ computation results in a mean difference of 2.5 Gy or 3.7% (Fig. 3a). After correction to equivalent 2‑Gy fractionation the mean difference becomes 73.4 Gy as the dose-per-fraction correction with the LQ model overestimates the EUD_CFRT_ by a factor of 1.5 compared to the USC model on average (Fig. 3b). Most of the outliers of LQ-EUD_CFRT_ are attributed to the NCI-H661 (*α/β*= 1.78 Gy) and the NCI-H569 (*α/β*= 3.91 Gy) cell lines and are associated with dose distributions having minimum dose points >57.5 Gy. In contrast, only the NCI-H661 cell line attributes to the outlying USC-EUD_CFRT_ values that depend on the minimum dose as well as the mean dose.

Fig. 3 also shows the box and whisker plots of the predicted LPFS. The resulting median USC-TCP and LQ-TCP are 93.8% and 99.4%, respectively.

**Fig. 4:**
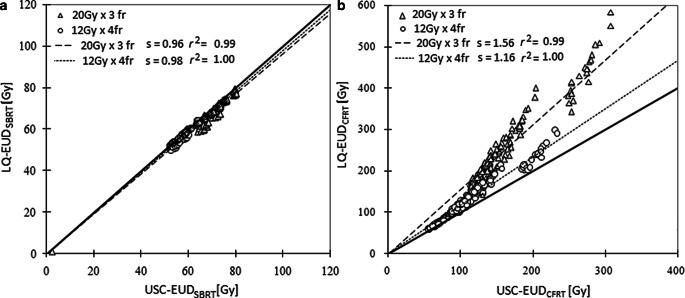
Plots of the USC-EUD against the LQ-EUD of SBRT dose-per-fraction (**a**) and of 2 Gy per fraction (**b**) delivered in two dose fractionation regimes (20 Gy × 3 fractions and 12 Gy × 4 fractions). The *straight line* with a slope of 1 represents the equivalent line where the USC-EUD_SBRT_ (USC-EUD_CFRT_) equals the LQ-EUD_SBRT_ (LQ-EUD_CFRT_). The other lines represent the best fit to the data with a zero intercept; *s* represents the slope and *r*^*2*^ the coefficient of determination of the linear regression

**Fig. 5:**
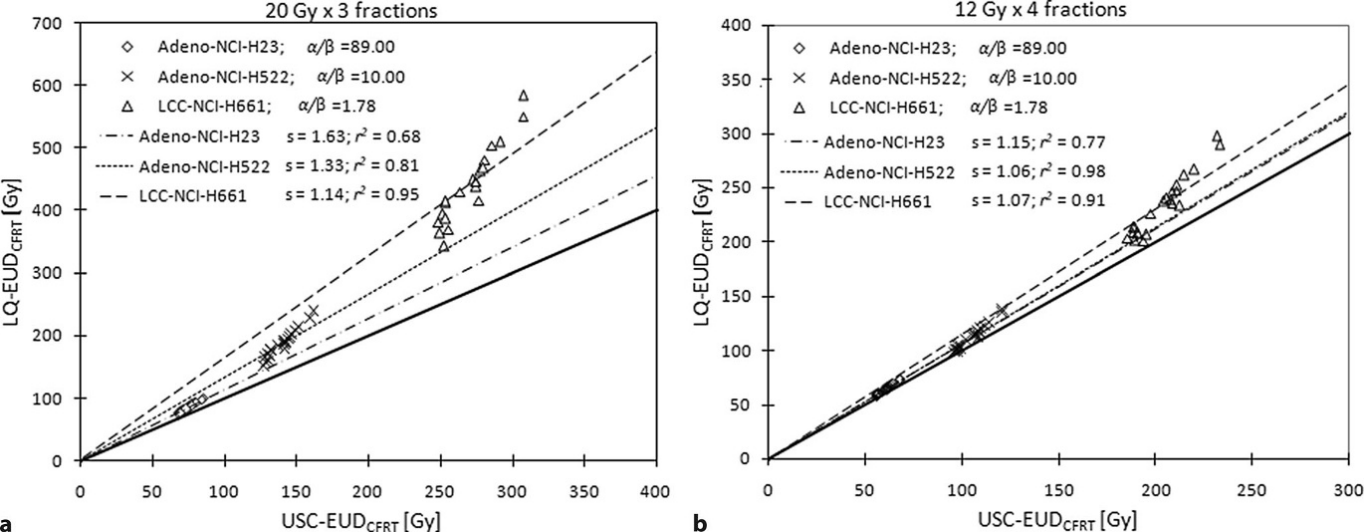
Plots of the USC-EUD_CFRT_ against the LQ-EUD_CFRT_ for different $\alpha /\beta$ ratios (denoted as *α/β*= 89.00, 10.00, and 1.78) for **a** 20 Gy × 3 fractions and **b** 12 Gy × 4 fractions. The *straight line* with a slope of 1 represents the equivalent line where the USC-EUD_CFRT_ equals the LQ-EUD_CFRT_. The other lines represent the best fit to the data with a zero intercept; *s* represents the slope and *r*^*2*^ the coefficient of determination of the linear regression

Since lung SBRT is delivered via different fractionation schemes, the above calculations were repeated assuming a total dose of 48 Gy delivered in four fractions to evaluate the resulting effects. The relationship between EUD values derived using the LQ and USC models is illustrated in Fig. 4. A linear regression of the data (with zero intercept) results in the following relationships; LQ-EUD_SBRT_ = 0.96 × USC-EUD_SBRT_ (*r*^2^ = 1.00) and LQ-EUD_CFRT_ = 1.56 × USC-EUD_CFRT_ (*r*^2^ = 0.99) for 60 Gy in three fractions, and LQ-EUD_SBRT_ = 0.98 × USC-EUD_SBRT_ (*r*^2^ = 1.00) and LQ-EUD_CFRT_ = 1.16 × USC-EUD_CFRT_ (*r*^2^ = 1.00) for 12 Gy × 4 fractions, respectively. All correlations were significant with a *p* < 0.05. The fits to the data for LQ-EUD_CFRT_ vs. USC-EUD_CFRT_ deteriorate from 12 Gy × 4 fractions to 20 Gy × 3 fractions (Fig. 4) and from high to low *α/β* ratio (Fig. 5).

## Discussion

That the linear-quadratic (LQ) dose–effect relation is inappropriate to describe in vitro log cell survival data at a large dose per fraction is well documented [6]. As a result, equivalent uniform dose (EUD) values based on the LQ model may not be appropriate. We attempt to address this issue by adapting the EUD formalism to the universal survival cell (USC) model.

Using cell survival data of nine non-small cell lung carcinoma (NSCLC) cell lines in the literature, our data suggest that the USC-EUD formalism results in higher EUD_SBRT_ values than the LQ-EUD formalism in the presence of cold and hot spots in different fractional volumes. The deviation of the USC-EUD_SBRT_ from the LQ-EUD_SBRT_ is relatively small, of the order of 5%, but significant (*p* < 0.05) for all studied DVHs including the simplified two-compartment tumor models and the clinical cases. In general, the deviation is smaller if the dose variation represents a dose boost and larger otherwise. The deviation saturates and even declines at larger dose deficits, since the cell survival curve described by the USC approached that described by the LQ model as the fraction dose *d* decreased. The almost linear increase of LQ-EUD_SBRT_ with hot spot showed by Kavanagh et al. [18] also happened with USC-EUD_SBRT_.

The variation in EUD with radiosensitivity parameters—a direct consequence of the target-cell theory inherent to the concept of EUD—is unsurprising. As pointed out by Niemierko [26], EUD is more sensitive to *α* or SF_2_ if there is a very steep gradient of cell survival probabilities within the irradiated volume. It should be noted that most studies of EUD focused on conventional doses of 2 Gy per fraction, around which EUD should decrease faster for larger values of *α* [27, 28]. At high dose, the dose-rate-dependent parameter β starts to outweigh the dose-rate-independent parameter α to become dominant in determining the cell survival probability in the LQ dose–effect formalism. For the USC formalism, it is the final slope, −1/D_o_, that determines the cell killing. The reason that the calculated LQ-EUD_SBRT_ drops faster with higher β and USC-EUD_SBRT_ with lower D_o_ values can be explained via the mathematical interpretation of these parameters in the respective models. In the LQ model, the shoulder of the negative log cell survival does not bend downwards as much for low β values as for high β values. This means that cells with low β values are less radiosensitive at large acute doses. In the USC model, the slope of the linear portion of the cell survival curve*-dlnSF/dD* is larger for lower D_o_ values, meaning that target cells are more radiosensitive.

Since cell survival data were fitted using different models (e.g., single-/multi-target single hit, LQ, etc.), the resulting model parameters describing the cell radiosensitivity may not coincide exactly. In the nine cell lines examined, the NCI-H290 cells of the mesothelioma reportedly have a relatively low β value of 0.012 Gy^−2^ and a medium D_o_ value of 1.3 Gy. Entering these values into Eq. 10 and Eq. 12 would lead to different conclusions from the results. In the first instance a moderate sensitivity to dose variation for this cell line relative to other studied cell lines would be indicated but in the latter, a relative insensitivity of dose variation would be indicated. Such different dependency of the radiosensitivity parameters partly explains why differences between the USC-EUD_SBRT_ and LQ-EUD_SBRT_ do not increase or decrease in accordance with either β or D_o_.

Using clinical dose volume histogram (DVH) models, the two formalisms continue to show some deviations of the EUD_SBRT_ values that depend on the SBRT dose fractionation and the cell lines (Fig. 4). The linear fit to USC-EUD_SBRT_ and LQ-EUD_SBRT_ over all cell lines approaches the line of unity as the dose per fraction decreases from 20 Gy (for three fractions) to 12 Gy (for four fractions) because the slopes of the LQ and USC models converge towards the transition dose d_T_. This means that a calibration between USC-EUD_SBRT_ and LQ-EUD_SBRT_ can be established by a scaling factor of 0.96 and 0.98 for 20 Gy and 12 Gy per fraction, respectively. The clinical implication of using either EUD formalism for radiobiological dose assessment and dose reporting for the same dose fractionation may be insignificant for the fact that dose inhomogeneity in SBRT largely represents an increase of center dose. In such cases, the mean deviation of two EUD formalisms is limited to 0.5 Gy or 4% up to a fraction dose of 20 Gy over all cell lines.

As previously discussed by Jones and Hoban [21], both Eq. 10 and 12 are limited to the same number of fractions for EUD as the inhomogeneous dose distribution and therefore do not account for the change of biological effect with dose per fraction and total dose. This study adapted the concept of biological equivalent uniform dose (BEUD) [21] and the normalized EUD in 2‑Gy fractions (i.e., EUD_CFRT_) to account for the difference in the dose potency of SBRT treatments delivered in different fractionation schemes. Although the difference of LQ- and USC-EUD_SBRT_ is relatively small at the non-standard SBRT dose fractionations, it was seen that the potency of EUD_SBRT_ at a large dose per fraction was grossly overpredicted by the LQ model, resulting in significantly larger EUD_CFRT_ values by a factor of 1.46 and 1.14 over all cell lines for 60 Gy in three fractions and 48 Gy in four fractions, respectively, compared to the USC model. A similar factor of 1.48 was estimated for the LQ-BED vs. USC-BED given a uniform dose of 20 Gy to the NCI-460 cell line in vitro by Park et al. [6]. This overprediction of dose potency was indeed the center of debate over the validity of LQ at high dose in recent years. This study demonstrated further that the magnitude by which the LQ model overpredicts the dose potency primarily depends on the fitting parameters of cell survival models but is relatively insensitive to the characteristics of the underlying dose distributions typical of clinical SBRT with ~15 to 45% dose increase. This is because the deviation of EUD_CFRT_ between the two formalisms is almost constant for each cell line given 10 to 30% dose increase above the prescription with little volume dependency, as illustrated in the two-compartment DVH models (Table 2). In fact, the regressions of LQ-BED/NTD and USC-BED/NTD yield the same slopes and *r*^*2*^ as those obtained for the clinical LQ- and USC-EUD_CFRT_ in Fig. 4_._ (Supplementary Fig. S1). Furthermore, our regression results also suggest that a calibration between LQ-EUD_CFRT_ and USC-EUD_CFRT_ can be established per dose fractionation scheme, acknowledging that the prediction errors would be larger in cell lines with lower *α/β* ratios but reduced with decreasing the dose per fraction, as shown in Fig. 5.

When all values for the dose distributions exceed the transition dose d_T_, either Eq. 10 or Eq. 12 alone suffices for computing the EUD. But for dose distributions having dose points less than and greater than d_T_, Eq. 10 and Eq. 12 have to be invoked simultaneously. This obviously complicates the EUD computation. More importantly, the number of model parameters increases from one to three with our proposed USC-EUD formalism. Indeed, other functional forms may be appropriate provided that they do equally well at describing or fitting the cell survival data over the full dose range. For example, the EUD can be derived from the McKenna–Ahmad variation of the repair model [10]. While the USC, McKenna–Ahmad model [10], and others [18] offer apparently equally good fits, a limitation to adopting these alternative formalisms would be expected to lie in the uncertainty and variation in the cell radiosensitivity parameters. Also, every model requires its own parameters (e.g., the K_g_ and K_o_ parameters of Kavanagh and Newman [29]).

It is worth noting that this study concentrated on the effect of incorporating a better description of cell survival in the EUD formalism. The inclusion of the absolute volume of the tumor, proliferation effect, and inhomogeneity of the patient population are logical continuations. It is also important to note that any calculated EUD values depend on the dose calculation algorithm used to obtain the dose distribution.

In a single-institutional study, McCammon et al. reported a significant difference in actuarial local control between three EUD levels [30]. Based on dose distributions calculated with a similar type‑A dose algorithm that did not account for charged particle disequilibrium, they reported 3‑year local control of 89.9% in the highest EUD group of >65.3 Gy. In their study, the EUD was calculated using the formalism based on the cell survival fraction at a reference dose of 2 Gy (SF_2_) with no consideration of the dose-per-fraction effect [31]. In this study, the calculated values for the LQ-EUD_CFRT_ predict a 3-year local progression-free survival (LPFS) rate of almost 100.0%. In contrast, using the USC-EUD_CFRT_ values would yield a more reasonable LPFS estimation (median 93.8%) but still higher than the median of 56.5% (range 95.9–46.0%) reported in the literature [32–38]. Such deviation of predicted outcome from actual clinical data is likely due to the model parameters used to estimate the LPFS and the slightly more potent dose regime (20 Gy for three fractions) used in this study. It is worth noting that the TCP outliers (Fig. 3c) were inverse of the EUD_CFRT_ outliers (Fig. 3b). This was because the cell lines with lower *α/β* ratios had larger EUD_CFRT_ values, which in turn translated into better TCP. The reverse applies to the results in cell lines with higher α/β ratios and hence lower TCP values due to lower EUD_CFRT_ values.

In a recent TCP modeling study, Santiago et al. pooled the local control results from the literature and applied the LQ and the linear-quadratic-linear (LQL) model to fit these data [16]. They concluded no obvious advantage of one model over the other as assessed by the goodness of fit. Without detailed knowledge of the planned dose distribution and dose–volume histograms, their study had to assume a uniform dose at the prescription level across the tumor volume. Nonetheless, Guckenberger et al. later demonstrated much better correlation of the local tumor control with the approximate maximum dose at the isocenter than the minimum doses at the planning target volume (PTV) periphery dose (approximate minimum dose) without significant difference between the LQ and LQL formalisms [15]. In a more recent study, Shuryak et al. argued that the LQ model including heterogeneous tumor radiosensitivity alone without introduction of additional extra-high dose terms as in the USC model achieved the best fit to the pooled clinical data, regardless of modeling dose to the isocenter or to the tumor periphery [39]. The insignificant difference in the dose–response relationship modeled by the LQ and other formalisms such as the USC-based on simple DVH metrics (e.g., approximate maximum and minimum doses at isocenter and PTV periphery) may also be expected with the EUD because of the almost constant deviation between the LQ- and USC-EUD_CFRT_ across the high dose gradient. Yet, the EUD may still be a useful tool to reveal the inconsistent results on the role of the high dose gradient in SBRT in the dose–response relationship.

## Conclusion

For EUD to be a useful tool for reporting SBRT dose with a high dose gradient within the target volume, a unified formalism should be defined among the SBRT community because its value depends on the underlying radiobiological model and the model parameters. Further investigations of the optimal formalism to derive the EUD through clinical correlations are warranted.

## Caption Electronic Supplementary Material

Fig.S1. Plot of the deviations between the linear-quadratic (LQ) and universal survival curve (USC) models in calculating the biological effective dose (and normalized total dose in 2‑Gy fractions) for a uniform dose distribution vs. equivalent uniform dose (EUD) of the clinical SBRT dose distributions of the same prescription dose. Black and gray symbols represent results of 20 Gy for three fractions and 12 Gy for four fractions, respectively. Linear regressions with zero intercept resulted in a slope of 0.98 and coefficient of determination r^2^ of 1 for both dose fractionation schemes.
