# Supplementary material for: Revisiting the formalism of equivalent uniform dose based on the linear-quadratic and universal survival curve models in high-dose stereotactic body radiotherapy
Source: Strahlenther Onkol. 2020 Nov 27;197(7):622–32. doi: 10.1007/s00066-020-01713-w (PMC8219592; doi:10.1007/s00066-020-01713-w)
Supplement: Supplementary file 1 — Fig.S1. Plot of the deviations between the linear-quadratic (LQ) and universal survival curve (USC) models in calculating the biological effective dose (and normalized total dose in 2‑Gy fractions) for a uniform dose distribution vs. equivalent uniform dose (EUD) of the clinical SBRT dose distributions of the same prescription dose. Black and gray symbols represent results of 20 Gy for three fractions and 12 Gy for four fractions, respectively. Linear regressions with zero intercept resulted in a slope of 0.98 and coefficient of determination r2 of 1 for both dose fractionation schemes. [file 66_2020_1713_MOESM1_ESM.docx]

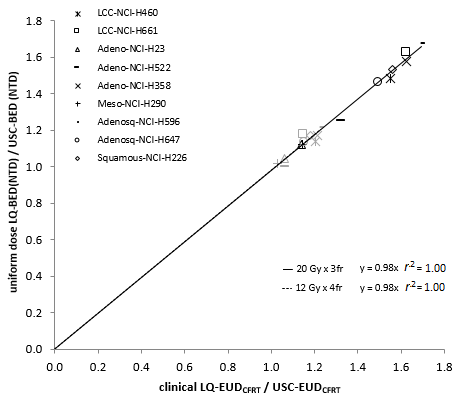


Fig.S1. Plot of the deviations between the linear-quadratic (LQ) and universal survival curve (USC) models in calculating the biological effective dose (and normalized total dose to 2 Gy fractions) for a uniform dose distribution vs. equivalent uniform dose (EUD) of the clinical SBRT dose distributions of the same prescription dose. Black and gray symbols represent results of 20 Gy for 3 fractions and 12 Gy for 4 fractions, respectively. Linear regressions with zero intercept resulted in a slope of 0.98 and coefficient of determination *r^2^* of 1 for both dose fractionation schemes.
